# *Brassica oleracea* var. *acephala* (kale) improvement by biological activity of root endophytic fungi

**DOI:** 10.1038/s41598-020-77215-7

**Published:** 2020-11-19

**Authors:** Jorge Poveda, Iñigo Zabalgogeazcoa, Pilar Soengas, Victor M. Rodríguez, M. Elena Cartea, Rosaura Abilleira, Pablo Velasco

**Affiliations:** 1 Biological Mission of Galicia (CSIC), Pontevedra, Spain; 2 Institute of Natural Resources and Agrobiology of Salamanca (CSIC), Salamanca, Spain

**Keywords:** Microbiology, Applied microbiology, Microbial communities, Fungi, Plant sciences, Plant stress responses, Plant symbiosis

## Abstract

*Brassica oleracea* var. *acephala* (kale) is a cruciferous vegetable widely cultivated for its leaves and flower buds in Atlantic Europe and the Mediterranean area, being a food of great interest as a "superfood" today. Little has been studied about the diversity of endophytic fungi in the *Brassica* genus, and there are no studies regarding kale. In this study, we made a survey of the diversity of endophytic fungi present in the roots of six different Galician kale local populations. In addition, we investigated whether the presence of endophytes in the roots was beneficial to the plants in terms of growth, cold tolerance, or resistance to bacteria and insects. The fungal isolates obtained belonged to 33 different taxa. Among those, a *Fusarium* sp. and Pleosporales sp. A between *Setophoma* and *Edenia* (called as *Setophoma*/*Edenia*) were present in many plants of all five local populations, being possible components of a core kale microbiome. For the first time, several interactions between endophytic fungus and *Brassica* plants are described and is proved how different interactions are beneficial for the plant. *Fusarium* sp. and Pleosporales sp. B close to *Pyrenophora* (called as *Pyrenophora*) promoted plant growth and increased cold tolerance. On the other hand, isolates of *Trichoderma* sp., Pleosporales sp. C close to *Phialocephala* (called as *Phialocephala*), *Fusarium* sp., *Curvularia* sp., *Setophoma*/*Edenia* and *Acrocalymma* sp. were able to activate plant systemic resistance against the bacterial pathogen *Xanthomonas campestris*. We also observed that *Fusarium* sp., *Curvularia* sp. and *Setophoma*/*Edenia* confered resistance against *Mamestra brassicae* larvae.

## Introduction

*Brassica* crops represent one of the 10 most economically important vegetables in world agricultural and food markets. The most important crop species in this genus are *Brassica oleracea* (i.e. cauliflower, broccoli, Brussels sprouts, kale, cabbage, …), *Brassica napus* (i.e. rapeseed and leaf rape), and *Brassica rapa* (i.e. turnip, Chinese cabbage, pak choi…), being mainly cultivated in temperate regions of the Northern Hemisphere^1^. Kale, *B. oleracea* var. *acephala*, is a leafy vegetable which has gained a great popularity as a “superfood” in recent years, due to its anticancerogenic and antioxidant potential associated with the presence of various compounds from the polyphenol, glucosinolate, terpenoid or carotenoid group, and contents of Ca, folate, riboflavin, vitamin C, K and A^2^. Furthermore, kale is an important vegetable crop in Iberian Peninsula traditional farming systems, grown for their leaves and flower buds^3^.

The role of microbes in determining the health of soils and plants is increasingly acknowledged. Plants harbour a diversity of microorganisms that may engage in a continuum of interactions ranging from beneficial to adverse interactions. Some of these interactions may be transient and occur during a specific life stage of the plants regardless of its beneficial, detrimental or neutral impact^4^. Endophytes are microbes that can be isolated from asymptomatic plant tissue, including neutral, commensal and/or beneficial microorganisms as well as dormant saprobes and latent pathogens^5^. Some fungal endophytes are well known to contribute to plant fitness, improving the host adaptation to biotic and abiotic stress conditions^6^. For instance, some endophytes are able to reduce the damage of plant pathogens thanks to antagonism via hyperparasitism, competition or antibiosis, or by means of the activation of plant defences^7,8^; others act as entomopathogens (e.g. *Beauveria bassiana* or *Metarhizium anisopliae*), protecting host plants against herbivore pests by direct exposure or through the production of insecticidal compounds^9^. Moreover, endophytic fungi are able to increase the plant’s tolerance against abiotic stress factors such as drought, salinity or high temperature through the activation of host stress-responses, allowing the plants to avoid or mitigate the impact of the stress^10^.

As far as the *Brassica* genus is concerned, there are some studies on the diversity of endophytic fungi and their possible role in improving plant productivity^11^, although none has been done in kale. The inoculation of *B. napus* with *Piriformospora indica* promoted plant growth, seed yield and quality^12^. Several works focused on the impact of endophytic fungi in the resistance against biotic stresses. *Aspergillus flavipes*, *Chaetomium globosum*, *Clonostachys rosea* and *Leptosphaeria biglobosa* isolated from *B. napus* suppressed leaf blight of oilseed rape caused by *Sclerotina sclerotiorum*^13^. *Aspergillus capensis* had antifungal activity against the plant pathogenic fungi *Botrytis cinerea*, *Monilinia fructicola*, *Sclerotinia trifoliorum* and *S. sclerotiorum*^14^. Moreover, the inoculation of *B. napus* with a *Metarhizium anisopliae* endophyte provided the plants with greater resistance against *Plutella xylostella* larvae^15^.

Compared to other plant species, it is considered that the diversity of endophytic fungi present in brassicas is lower than that of other taxonomic groups, probably because of the presence of secondary metabolites derived from tryptophan and other aminoacids, known as glucosinolates^16^. Despite this, the use of endophytic fungi could have an impact on cultivation and the improvement of *Brassica*-crops within a more environmentally friendly agriculture.

On the other hand, the diversity of endophytic fungi associated with different species within the same genus can be abysmal, and even within the varieties of the same species, as already observed in *Uncaria gambier* gambir udang and gambir nasi varieties^17^, or in *Rosa multiflora* and var. *carnea*^18^.

At present, there are no diversity studies of endophytic fungi present in the roots of *B. oleracea* and very few in other species of the genus. Therefore, the main objective of this study was to estimate the diversity of root endophytes associated with different accessions of *kale.* Secondly, we evaluated the biological effect of some dominant endophytes in the promotion of plant growth, tolerance against cold, and resistance against pests and diseases.

## Results

### Fungal diversity

From the 900 root-fragments (180 per *B. oleracea* accession), 376 fungal isolates were obtained, at a rate of 54–98 fungal isolates per accession. Isolates were obtained from 41.77% of the root fragments plated. All sampled plants harbored fungi in their roots, and on average, 13 isolates were obtained from the roots of each plant.

The isolates were grouped into morphotypes, obtaining 27 morphotypes for MBG-BRS0106, 27 for MBG-BRS0292, 45 for MBG-BRS0426, 33 for MBG-BRS0446 and 21 for MBG-BRS0468. After sequencing one or more isolates of each morphotype, 179 ITS1-5.8S-ITS2 nucleotide sequences were obtained. After those sequences were clustered, considering that those being 97% or more identical belonged to the same taxon, 33 different fungal taxa were identified (Table 1). A phylogenetic tree made with the sequences of these 33 taxa helped to accommodate into taxonomic orders isolates whose sequences were less than 95% identical to those of a type strain (Supplementary Fig S1).

**Table 1:** Core and abundant fungal species isolated from surface sterilized roots of *B. oleracea* (kale) from 5 different accessions.

Type strain with closest sequence identity	Identity to closet match (%)	Proposed taxon	ITS sequence accession number	Order	Incidence in plants (%)	Number of *B. oleracea* accesions
*Fusarium foetens*	99.3	*Fusarium* sp.	MT628384	Hypocreales	63.3	5
*Edenia gomezpompae*	92.5	*Pleosporales* sp. A	MT628351	Pleosporales	56.7	5
*Acrocalymma fici*	95.9	*Acrocalymma* sp.	MT626728	Pleosporales	46.7	3
*Alternaria destruens*	100	*Alternaria* sp. A	MT628452	Pleosporales	20.0	3
*Pyrenophora nisikadoi*	94.7	*Pleosporales* sp. B	MT628399	Pleosporales	13.3	3
*Alternaria mimicula*	100	*Alternaria* sp. B	MT628543	Pleosporales	13.3	3
*Barrenia panicia*	98.5	*Barrenia* sp.	MT636549	Helotiales	13.3	3
*Ceratobasidium ramicola*	93.1	Basidiomycota A	MT629733	unknown	10.0	1
*Phialocephala hiberna*	94	Helotiales sp.	MT628664	Helotiales	6.7	2
*Polyschema sclerotigenum*	98.4	*Polyschema* sp.	MT628702	Pleosporales	6.7	2
*Mucor moelleri*	99.1	*Mucor* sp. A	MT639934	Mucorales	6.7	1
*Curvularia coatesiae*	99.6	*Curvularia* sp.	MT640053	Pleosporales	6.7	1
*Cladosporium spp.*	99.8	*Cladosporium* sp.	MT641243	Capnodiales	6.7	2
*Phomopsis tuberivora*	99.4	*Diaporthe* sp	MT636064	Diaporthales	6.7	1
*Phoma schachtii*	99.2	*Phoma* sp.	MT628903	Pleosporales	3.3	1
*Rhizopus oryzae*	99.8	*Rhizopus* sp.	MT635401	Mucorales	3.3	1
*Mucor hiemalis*	96.2	*Mucor* sp. B	MT636070	Mucorales	3.3	1
*Penicillium cremeogriseum*	99.8	*Penicillium* sp.	MT636161	Eurotiales	3.3	1
*Chaetomium novozelandicum*	99.8	*Chaetomium* sp.	MT641231	Sordariales	3.3	1
*Trichoderma hamatum*	99.8	*Trichoderma* sp.	MT641233	Hypocreales	3.3	1
*Codinaea acaciae*	98.1	*Codinaea* sp.	MT640043	Chaetosphaeriales	3.3	1
*Dendryphion europaeum*	96	*Dendryphion* sp.	MT641239	Pleosporales	3.3	1
*Minutisphaera aspera*	99.4	*Minutisphaera* sp	MT636088	Minutisphaerales	3.3	1
*Ceratobasidium papillatum*	90.7	Basidiomycota B	MT640104	unknown	3.3	1
*Ceratobasidium ramicola*	91.1	Basidiomycota C	MT636101	unknown	3.3	1
*Phragmocephala garethjonesii*	92.8	*Pleosporales* sp. C	MT641235	Pleosporales	3.3	1
*Trametes versicolor*	99.4	*Trametes* sp.	MT635595	Polyporales	3.3	1
*Aaosphaeria arxii*	99	*Aaosphaeria* sp.	MT645080	Pleosporales	3.3	1
*Mycofalcella calcarata*	96.9	*Mycofalcella* sp.	MT636550	Helotiales	3.3	1
*Aspergillus aureolus*	99.6	*Aspergillus* sp. B	MT639933	Eurotiales	3.3	1
*Aspergillus *spp*.*	98.4	*Aspergillus* sp. A	MT641240	Eurotiales	3.3	1
*Tetraploa sasicola*	95.6	*Tetraploa* sp.	MT641232	Pleosporales	3.3	1
*Plectosphaerella niemeijerarum*	99.4	*Plectosphaerella* sp.	MT641266	Glomerellales	3.3	1

A core microbiome is defined as the group of microbes commonly found within a host's microbiome, using persistence of the association as the criterion to select microbes potentially providing critical function within the habitat in which they are found^67^.

**Figure 1:**
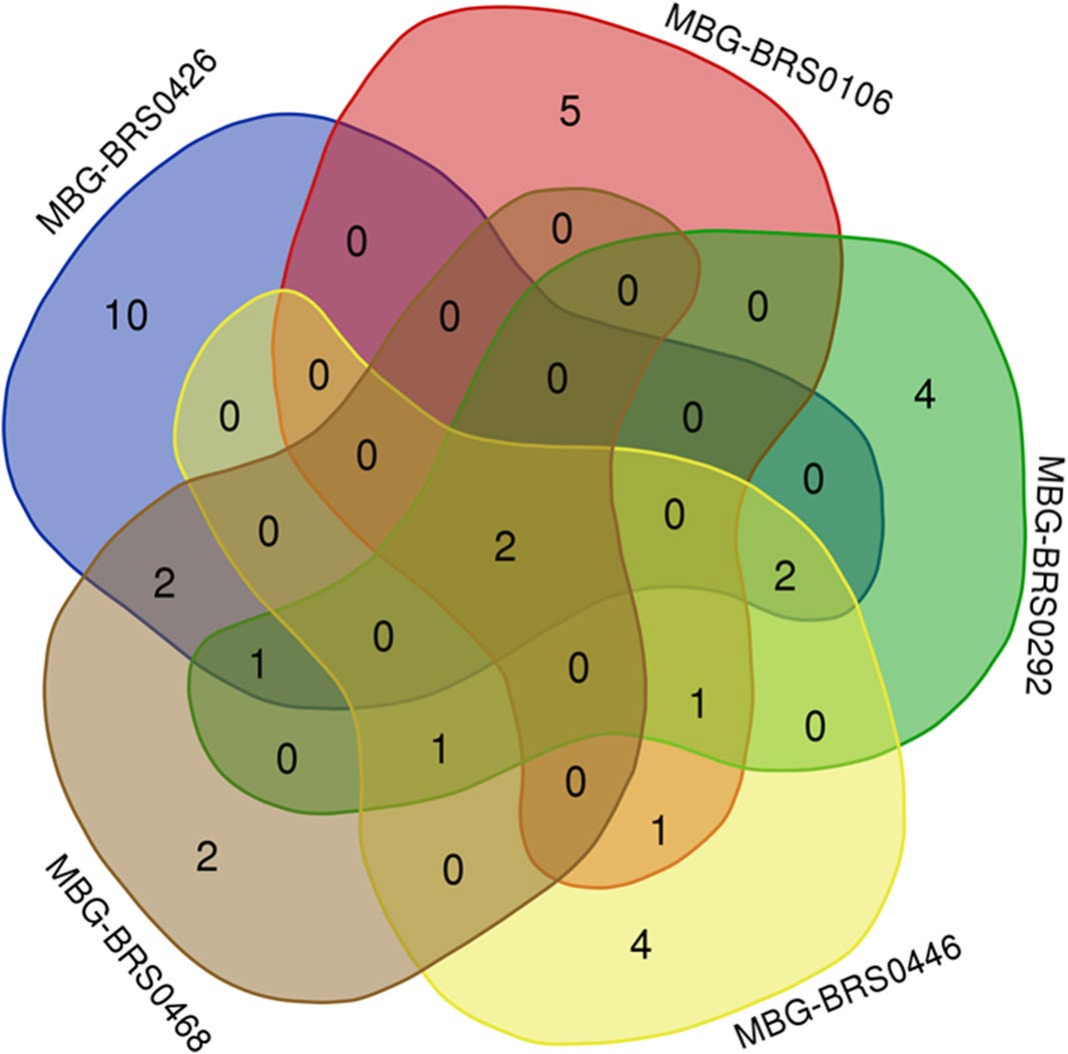
Venn diagram showing the distribution of fungal taxa isolated from the different accessions of *B. oleracea* (kale).

The most prevalent taxa were a *Fusarium* sp. found in 63.3% of the plants collected, and *Pleosporales* sp. A, found in 56.7%, both were present in all *B. oleracea* accessions (Table 1). Thanks to the creation of the phylogenetic tree and the comparison of the representative sequence of the proposed taxon *Pleosporales* sp. A, we have determined how this sequence represents a species not yet described, between the genus *Edenia* and *Setophoma* (called from now *Setophoma*/*Edenia*). The distribution of the fungal taxa showed few commonalities among the kale accessions (Fig. 1). MBG-BRS0426 and MBG-BRS0468 have 5 fungal taxa in common, 2 of which were not shared with any other accession. Several taxa occurred in only one accession: 10 taxa in MBG-BRS0426, 5 taxa in MBG-BRS0106, 4 taxa in MBG-BRS0292, 4 taxa in MBG-BRS0446, and 3 taxa in MBG-BRS0446. The similarity analysis based on the Jaccard index (JI) (Table 2), shows that only the accessions MBG-BRS0292 and MBG-BRS0446 present a similarity between their fungal taxa greater than 0.3, while above 0.25 we find MBG-BRS0292 and MBG-BRS0468, MBG-BRS0426 and MBG-BRS0468, and MBG-BRS0446 and MBG-BRS0106.

**Table 2:** Jaccard index of similarity (bold) and total number of fungal taxons identified in roots of each pair of *B. oleracea* (kale) accessions.

*B. oleracea* accession	MBG-BRS0426	MBG-BRS0106	MBG-BRS0292	MBG-BRS0446	MBG-BRS0468
MBG-BRS0426	**1.000**	**0.083**	**0.200**	**0.083**	**0.250**
MBG-BRS0106	24	**1.000**	**0.176**	**0.250**	**0.133**
MBG-BRS0292	25	17	**1.000**	**0.375**	**0.267**
MBG-BRS0446	24	16	16	**1.000**	**0.188**
MBG-BRS0468	20	15	15	16	**1.000**

**Figure 2:**
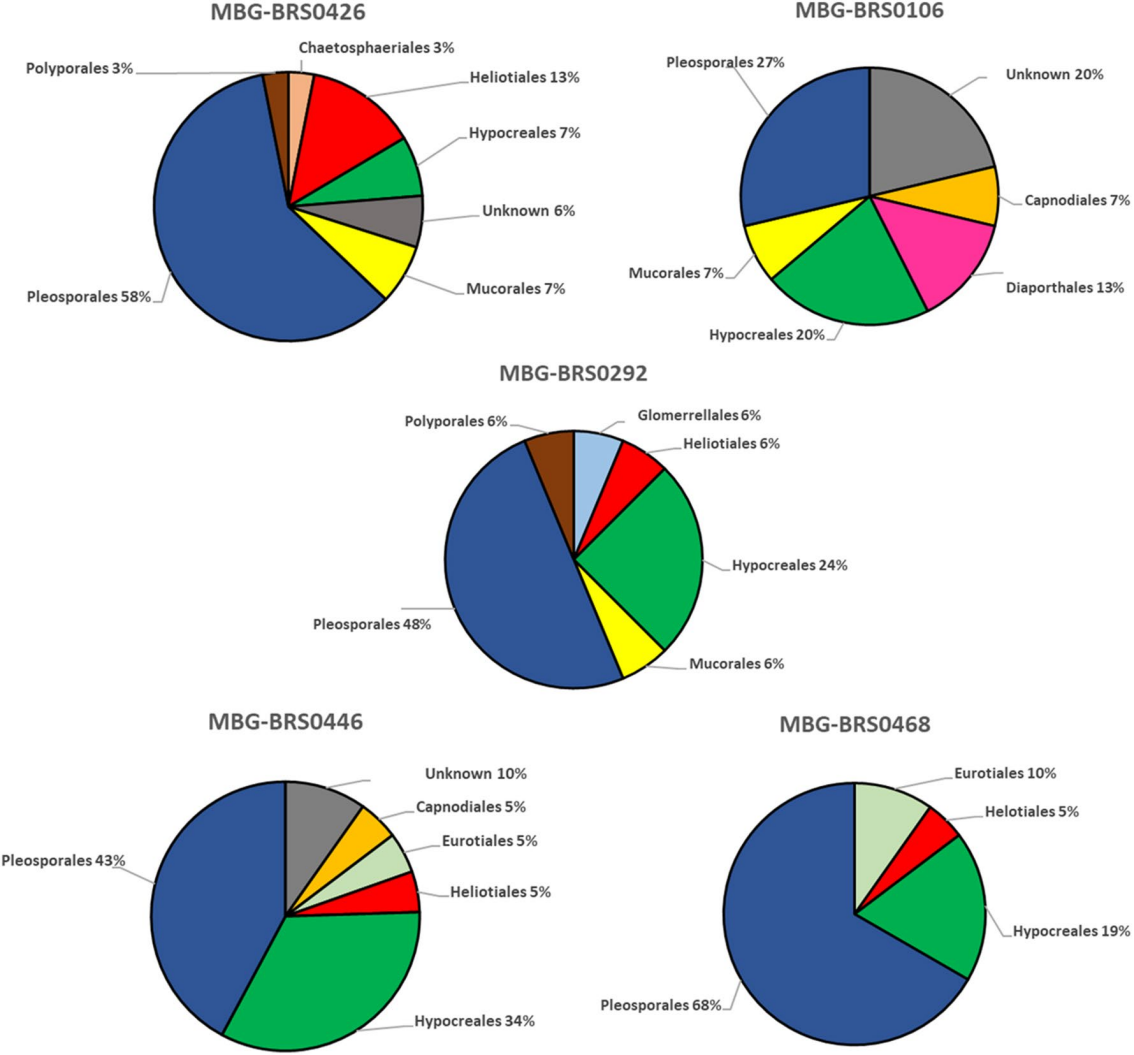
Distribution of fungal taxa from roots of different accessions of *B. oleracea* (kale) according to orders.

**Figure 3:**
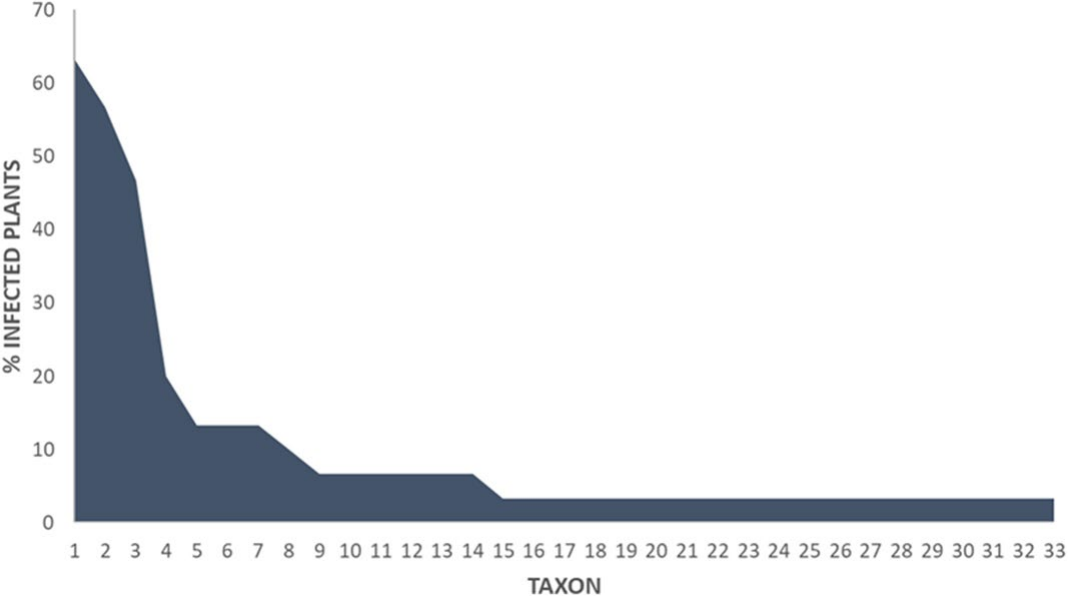
Rank-abundance plot showing the incidence in plants of each taxon identified in roots of *B. oleracea* (kale).

Pleosporales is the most representative order in terms of the number of taxa (32.3%) (Table 1). Most taxa are present only in one of the accessions (58.8%), with only 5.9% of the taxa present in all (Table 1). Taking into account the incidence of each order in each variety (Fig. 2), it can be seen that the most abundant order is Pleosporales (27–68%), followed by Hypocreales (7–34%), both present in all varieties. Helotiales is present in all accessions, except in MBG-BRS0106, where we find Diaporthales as an specific order. On the other hand, the distribution of taxa according to their incidence can be visualized in the rank-abundance curve shown in Fig. 3. Three taxa were present in more than 45% of the plants (*Fusarium* sp. [63.3%], *Setophoma*/*Edenia* [56.7%] and *Acrocalymma* sp.[46.7%]) while the remaining taxa were present in less than 15% of the plants (Table 1).

### Increased plant growth, tolerance to cold and resistance to biotic stresses

**Figure 4:**
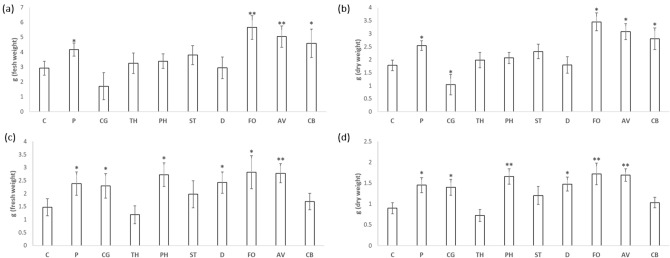
Mean of fresh (**a**,**c**) and dry weight (**b**,**d**) of kale plants grown in greenhouse (**a**,**b**) and cold (**c**,**d**) conditions. Plants without inoculation (C) and inoculated with unknown *Pyrenophora* (P), *Chaetomium* sp. (CG), *Trichoderma* sp. (TH), *Phialocephala* (PH), *Setophoma*/*Edenia* (ST), *Diaporthe* sp. (D), *Fusarium* sp. (FO), *Acrocalymma* sp. (AV) and *Curvularia* sp. (CB) were collected at 12-week-old and measured their fresh weight of the aerial part. Data are the mean of 25 plants for each inoculation with the corresponding standard deviation. Student’s t-test was performed. Asterisks denote significant differences at *P* ≤ 0.05 (*) and *P* ≤ 0.01 (**).

Nine fungal isolates were used to study beneficial effects on kale. Significant differences in the growth of the aerial part of kale were observed with the application of *Fusarium* sp. (5.66 g) and *Acrocalymma* sp. (4.83 g), being their weight almost double that observed for the control plants without inoculation (2.92 g). Growth of plants inoculated with *Pleosporales* sp. B next to *Pyrenophora* (called from now *Pyrenophora*) and *Curvularia* sp. was also significantly higher than that of the control plants. Likewise, although there was no significant difference respect to the control, we observed a reduction in the weight of the plants inoculated with *Chaetomium* sp. (1.71 g) (Fig. 4a); as well as dry weight (Fig. 4b).

Under cold conditions (constant 12 °C) is observed how inoculation with *Pyrenophora*, *Chaetomium* sp., Helotiales sp.next to *Phialocephala* (called from now *Phialocephala*), *Diaporthe* sp., *Fusarium* sp. and *Acrocalymma* sp*.* caused a significant increase in growth, and therefore, of tolerance to abiotic stress. Inoculation with *Phialocephala*,* Fusarium* sp. and *Acrocalymma* sp*.* increased the plant weight almost twice (2.72 g, 2.82 g, and 2.78, respectively) respect to uninoculated control plants (1.47 g) (Fig. 4c); as in dry weight (Fig. 4d).

**Figure 5:**
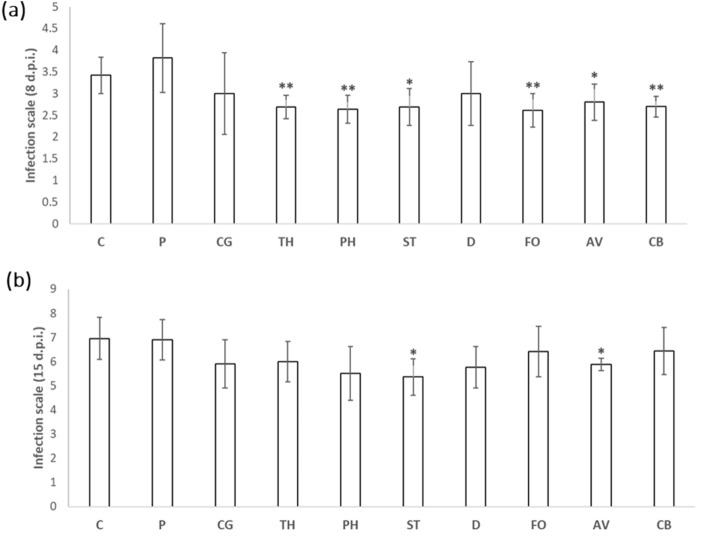
Effect of *Xcc* infection on kale plants inoculated with endophytic fungi. Plants without inoculation (C) and inoculated with unknown *Pyrenophora* (P), *Chaetomium* sp. (CG), *Trichoderma* sp. (TH), *Phialocephala* (PH), *Setophoma*/*Edenia* (ST), *Diaporthe* sp. (D), *Fusarium* sp. (FO), *Acrocalymma* sp. (AV) and *Curvularia* sp. (CB) were infected at 5–6 leaves stage and the leaves with lesions are classified according to an infection range from 1 to 5. Data are the mean 10 plants for each inoculation with the corresponding standard deviation. Student’s t-test was performed. Asterisks denote significant differences at *P* ≤ 0.05 (*) and *P* ≤ 0.01 (**).

Regarding the activation of systemic resistance against biotic stressors, leaves of *B. oleracea* were inoculated with the pathogenic *Xcc* bacteria (Fig. 5). We observed a significant decrease in the damage caused by the bacteria at 8 d.p.i (Fig. 5a) in the plants inoculated with *Trichoderma* sp., *Phialocephala*, *Fusarium* sp., *Curvularia* sp., *Setophoma*/*Edenia* and *Acrocalymma* sp*.*, being highly significant (P < 0.01) with the inoculation of the first four fungi indicated. On the other hand, at 15 d.p.i. (Fig. 5b) we only obtained a significant reduction in the incidence of the disease in the plants with the inoculation with the *Setophoma*/*Edenia* and *Acrocalymma* sp*.* strains.

**Figure 6:**
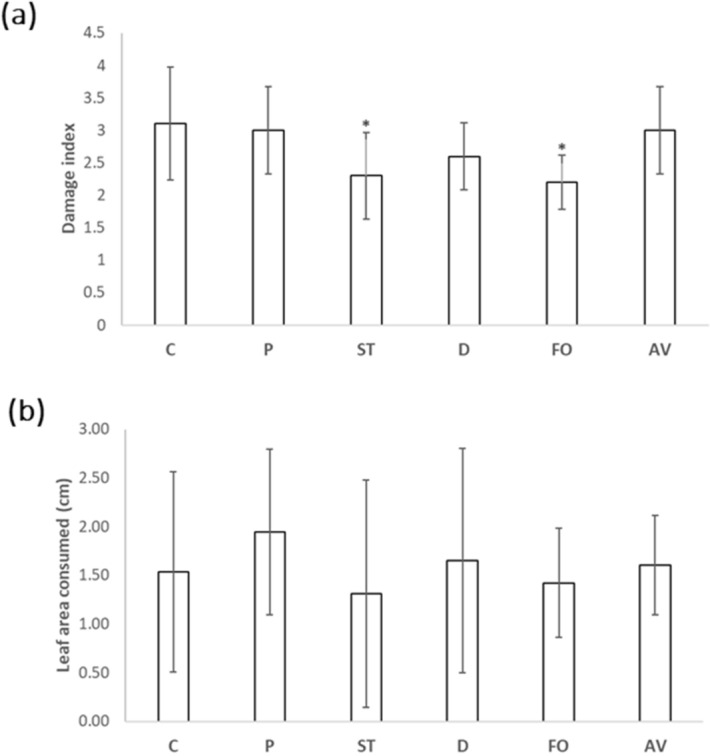
Effect of infestation with *M. brassicae* on kale plants inoculated with endophytic fungi. Plants without inoculation (C) and inoculated with *Pyrenophora* (P), *Setophoma*/*Edenia* (ST), *Diaporthe* sp. (D), *Fusarium* sp. (FO) and *Acrocalymma* sp. (AV) were attacked at 12-week-old, obtaining 5 d.p.a. the damage index in a range from 1 to 5 and the leaf area consumed by the larvae. Data are the mean of 10 plants for each inoculation with the corresponding standard deviation, and for each biological replicate and condition, three leaves/plants were used. Student’s t-test was performed. Asterisks denote significant differences at *P* ≤ 0.05 (*).

Regarding the possible activation of systemic resistance in the inoculated plants against the insect pest *Mb*, the inoculation of kale with the fungi *Setophoma*/*Edenia* (DI 2.3) and *Fusarium* sp. (DI 2.2) strains supposed a significant decrease in the damage index, compared to the control plants without inoculation (DI 3.1). Furthermore, although no significant differences were observed with respect to the leaf area consumed by the larvae, both *Setophoma*/*Edenia* and *Fusarium* sp. had the lowest average leaf area consumed (Fig. 6).

## Discussion

Few studies have investigated fungal diversity in varieties of the same plant species within a study site. In other words, whether genotypic variation affects the structure of the root microbiome. Regarding the *B. oleracea* accessions of our study, we have reported a possible specificity of accession. Most of the determined fungal taxa are only present in one accession and the similarity indices give great differences despite the fact that all the plants are in the same geographical plot. These differences could be due to differences in the root exudates produced by each kale accession, which could affect the ability of endophytic fungi to colonize the roots, just as it has been observed in different tomato genotypes and their rhizospheric microbiota^19^. Despite this, it is very difficult to consider the existence of such specificity in a study like this, since the possibility of detecting those fungi with low prevalence is more difficult than those that are present to a greater extent, also the great differences existing at the level of alpha (Table 1, Fig. 2) and beta (Table 2) microbial diversity. On the other hand, the presence in all accessions of *Fusarium* sp. and *Setophoma*/*Edenia*, suggests that those two taxa could be components of a core microbiome of kale. This could be confirmed by surveying kale root endophytes at different locations.

The diversity of endophytic fungi present in the roots of plants of the *Brassica* genus has been scarcely studied so far, as has the diversity of fungi between different varieties of the same species. In our study, most isolated fungi belonged to the Pleosporales order. To our knowledge, only one species of the Pleosporales order, *Leptosphaeria biglobosa,* has been found in *B. napus*^13^. Besides, order Hypocreales was found in all accessions. This order is more common in Brassicaceae, and taxa such as *Fusarium* sp.^20^, *C.* *rosea*^13^ and *M. anisopliae*^15^ have been reported in *B. napus*. Mucorales order was found in 3 of the 5 accessions, having previously being reported *Mucor* sp. in *B. campestris*^21^ and *B. chinensis*^22^. Sordariales order was only found in MBG-BRS0292 and MBG-BRS0468 accessions, having previously being described the fungus *Chaetomium globosum* in *B. napus* roots^13^. And Eurotiales order was only found in the MBG-BRS0446 accession, having previously reported the fungus *Penicillium* sp.^20^, *A. flavipes*^13^ and *A. capensis*^14^ in *B. napus* roots. Therefore, our study reports the first description of the Helotiales, Capnodiales, Diaporthales, Chaetosphaeriales and Minutisphaerales orders in roots of a plant of the *Brassica* genus; Heliotales order was widely present in 4 of the 5 accessions.

In our study carried out with different accessions of *B. oleracea* (kale), we have been able to determine the presence of two dominant taxa in all the accessions examined (*Fusarium* sp. and *Setophoma*/*Edenia*). Species of the genus *Fusarium*, such as *F. oxysporum* can be neutral, beneficial, or detrimental for host plants. *Fusarium* wilt is one of the most devastating diseases in agriculture, since some strains can also cause vascular wilts resulting in serious yield losses in affected crops. Despite this, numerous strains of *F. oxysporum* behave as endophytes capable of activating plant systemic resistance against the pathogenic strains of the fungus^23^, or produce protective secondary metabolites^24^, like other species such as *F. tricinctum*^25^. Nevertheless, all the existing studies with *F. oxysporum* f. sp. *conglutinans* describe it as a pathogen of the *Brassica* genus, for example, studying the transcriptome profiles in different lines of *B. rapa* after its inoculation^26^, or transcriptomic and proteomic studies in *B. oleracea*^27,28^. So, this is the first study that describes *Fusarium* as an endophyte of brassica roots, although *Fusarium oxysporum* and other species of the genus have been described as dominant components of the microbiome of several plant species^29^.

As regards the taxa *Setophoma*/*Edenia*, there is information about pathogenic and endophytic species in both genus. *Setophoma* includes disease-causing pathogenic fungal species such as leaf spot in *Camellia sinensis* (*S. antiqua*, S. longinqua, *S. yingyisheniae* and *S. yunnanensis*)^30^, but mainly to root level. *S. terrestris* (formerly called *Phoma terrestris*) causes pink root rot on members of the *Allium* genus and other crops, such as tomato, eggplant, pepper, maize and carrot. In 2017, the first case of pink root rot in the *Brassica* genus was described in *B. napus*^31^, being the only report of *S. terrestris*-*Brassica* interaction described so far. On the other hand, *S. terrestris* has been described as an endophyte of other plant species such as *Gloriosa superba*^32^ and horseradish (*Armoracia rusticana*), where is able to decompose various glucosinolates^33^. In turn, *Edenia* is a well-known genus of endophytic fungi that produce compounds of high medical interest such as anti-inflammatories^34^ or antileshmanials^35^. Furthermore, the *E. gomezpompae* species is capable of producing highly toxic compounds for plants, which are used as natural herbicides^36^. Both within the *Setophoma* genus and the *Edenia* genus, our isolate represents the first endophyte identified in roots of *Brassica* plants.

An *Acrocalymma* sp. was present in 3 of the 5 accessions studied. *A. medicaginis* has been described as a causal agent of crown and root rot in *Medicago* plants^37^. However, *A. vagum* is a dark septate endophyte able to decrease heavy metal content in tobacco^38^ and to promote plant growth in *Glycyrrhiza uralensis*^39^, but never before reported in *Brassica* plants.

Therefore, in the present study we have been able to identify several taxa of endophytic fungi in kale roots, which had not been described so far as endophytic fungi (although in some cases as pathogens) within the *Brassica* genus.

Once the different endophytic fungi in the kale roots were identified, we made a selection of them to determine their possible biological role in the plant. To do this, we inoculated kale plants and tried to determine if the endophytes were capable of promoting plant growth, increasing cold tolerance, or inducing systemic plant resistance against pathogens and/or pests.

The ability of different species of endophytes to promote plant growth as well as tolerance to abiotic stresses such as cold, or resistance to pathogens has been described^40^. In our study, we have observed that the *Pyrenophora* strain was capable of promoting the growth of the plant and its cold tolerance. This would be the first time that a benefit of the *Pyrenophora*-plant interaction has been described, this genus is best known for pathogens, such as *Pyrenophora teres*, causal agent of net blotch of barley^41^. The beneficial role observed in *B. oleracea* by *Pyrenophora* could be due to the defensive capacity of cruciferous plants through secondary metabolites such as glucosinolates, not present in other plant groups, which is why a possible pathogen in a species may be a harmless symbiont in *Brassica*. Moreover, inoculation with *Fusarium* sp. in *B. oleracea* also increased its growth and cold tolerance, more significantly than with *Pyrenophora*. Although there are numerous studies that demonstrate the ability of non-pathogenic strains of *Fusarium* to increase the resistance of plants against various pathogens, such as *F. oxysporum*^23^, their role as a possible promoter of plant growth or cold tolerance has not been studied.

The interaction *B. oleracea*-*Acrocalymma* sp. resulted in a significant increase in plant growth, something previously observed in other plants such as *Glycyrrhiza uralensis*^39^, *Medicago sativa* and *Ammopiptanthus mongolicus*^42^ in interaction with *A. vagum*. Similar results have been reported in *B. oleracea*-*Curvularia* sp. interaction, fungal genus that, although it includes important plant pathogens^43^, also has species classified as endophytes that promote plant growth, such as *C. geniculata* in *Parthenium hysterophorus* through phosphate solubilization and phytohormone production^44^.

Regarding the increase in cold tolerance, we have observed that the *B. oleracea*-*Chaetomium* sp. interaction results in a significant benefit for the plant. In this sense, several authors have described different mechanisms such as heat shock proteins or antioxidant compounds that, for example, the endophyte *C. globosum* has to tolerate cold, some of which may also be beneficial for the plant thanks to the interaction^45–47^. The interaction of *B. oleracea* with *Phialocephala* also produced an increase in plant tolerance to cold, as has been verified in different subarctic herbaceous plants by *Phialocephala fortinii*^48^. Also, we have observed the capacity of *Diaporthe* sp. and *Acrocalymma* sp. to increase the tolerance of *B. oleracea* to cold, being the first time, even within their fungal genera, that these beneficial capacities for plants have been described.

After infection with *Xcc*, we quantified a decrease in the harmful effect of the bacteria on *B. oleracea* plants preinoculated with *Trichoderma* sp. The ability of different *Trichoderma* species to activate plant systemic resistance against different pathogens has been widely proven in various plant species^49^, including *Brassica* plants as *B. napus* with *T. harzianum*^50,51^ or *T. viride*^52^, *B. rapa* with *T. pseudokoningii*^53^, and even *B. oleracea* var. *capitate* with *T. harzianum* against *Rhizoctonia solani*^54^.

As far as the *Fusarium* sp. strain is concerned, the ability of *F. oxysporum* endophytic strains to induce systemic plant resistance has been described in zucchini plants (*Cucurbita pepo*) against the insect *Aphis gossypii*^55^, in banana plants (*Musa* spp.) against the nematode *Radopholus similis*^56^, in *Asparagus officinalis* against the pathogen *F. oxysporum* f. sp. *asparagi*^57^, and in tomato plants against *F. oxysporum* f. sp. *lycopersici*^58^. While in the case of inoculation with *Phialocephala*, *Curvularia* sp., *Setophoma*/*Edenia* and *Acrocalymma* sp., the data obtained represents the first report of activation of plant systemic resistance by these fungi as root endophytes.

As conclusions, the diversity of endophytic fungi found in the kale roots of various accessions is lesser compared to non-brassicaceae species, where up to 49 fungal taxa can be found per plant^59^. In addition, several of the isolated fungal taxa have been widely described as pathogens of different crops, but never as endophytes, as species of the genus *Pyrenophora*, an aspect that occurs in kale probably due to their powerful defensive capacity through the use of glucosinolates against fungi^60^. On the other hand, we find taxa present in several of the accessions and even in all of them, as is the case of *Fusarium* sp. and *Setophoma*/*Edenia*, representing a possible core microbiome for kale. It is precisely these taxa that have shown in our preliminary studies a greater capacity to promote the productivity of kale, by promoting its growth, increasing its tolerance to cold and increasing its defensive capacity against pathogens and pests.

## Materials and methods

### Plant material, crop and sampling

Five kale accessions were used in this study. These are local populations from Galicia (Northwestern Spain), kept in the *Brassica* Germplasm Collection of the Misión Biológica de Galicia (MBG-CSIC). The codes for these accessions are MBG-BRS0106, MBG-BRS0292, MBG-BRS0426, MBG-BRS0446 and MBG-BRS0468.

The study was conducted in 2016 at MBG-CSIC (Galicia, NW Spain). Two hundred seeds of each accession were sown in trays in May 2016, and kept in a greenhouse under environmental conditions. After 50 days, when most seedlings were at the 4–5 leaf stage, one hundred and fifty plants of each accession were transplanted into an experimental plot randomly distributed. The distance was 0.8 m between rows and 0.6 m between plants. The experimental plot was established in a slightly acidic soil (pH close to 5.5), organic matter content of 6.3%, 100 ppm available P, 335 ppm assimilable K, 133 ppm changeable Mg, and 7.83 cmol/kg Ca^2+^. As management practices, no fungicide was applied, or any other pesticide, or any form of fertilization. To prevent the development of weeds, the soil was covered with a mesh that prevents their presence in the plot.

Sampling was carried out in 30-week-old plants, randomly choosing 6 plants per accession. After digging out the entire root system, samples of the roots were collected, and stored in cold for a few hours until processing in the laboratory.

### Isolation of fungi

A subsample of 30 root fragments of 4–5 cm per plant was collected. Disinfection of roots and fungal isolation was done following the procedure described by Ref.^29^. Each root sample was washed with tap water and then surface-disinfected with a solution of 20% commercial bleach (1% active chlorine) containing 0.02% Tween 80 (v:v) for 6 min, followed by treatment with an aqueous solution of 70% ethanol for 30 s. Finally, the roots were rinsed with sterile water and cut into pieces about 5 cm long. Thirty root pieces of each sample were plated in three Petri plates (10 pieces/plate) with potato dextrose agar (PDA) (Sigma-Aldrich, St. Louis, USA) containing 200 mg/L of chloramphenicol (the antibiotic was used to avoid the isolation of endophytic bacteria), and kept in the dark at room temperature. As mycelium emerged from a root fragment into the agar, a small piece of the mycelium from the leading edge of the colony was transferred to a new PDA plate and maintained at room temperature. The root fragment and remaining mycelium were taken out of the original plate to avoid overgrowth. The plates with root samples were checked daily for the presence of fungi for about 4 weeks.

### Identification of fungi and diversity analysis

The fungal isolates obtained from roots were first grouped into different morphotypes according to morphological characteristics such as colony color, exudate production, mycelium appearance, and growth rate^29^. One or a few isolates of each morphotype were used for further classification and identification based on rDNA nucleotide sequences. Fungal DNA was extracted from a small amount of mycelium scraped from a PDA culture using the Phire Plant Direct PCR Kit (Thermo Fisher Scientific). A ribosomal DNA region including the internal transcribed spacer 1 (ITS1), 5.8S rDNA, and ITS2 was amplified by PCR using primers ITS1 and ITS4^61^. Amplification conditions were: 98 °C for 5 min, followed by 35 cycles of 98 °C for 5 s, 54 °C for 5 s, and 72 °C for 20 s; after that the reaction was kept at 72 °C for 1 min. PCR amplicons were cleaned (MSB Spin PCRapace, Stratec biomedical, Germany) and sequenced at the Genomics Service in the CACTI, University of Vigo, Spain (https://cactiweb.webs.uvigo.es/).

All the sequences obtained were grouped into operational taxonomic units (OTU), considering that groups of sequences with a similarity greater than 97% belonged to the same OTU. This clustering operation was done using CD-Hit-Est software^62^. For taxonomic identification, a representative sequence of each OTU was used to search the GenBank nucleotide database using BLAST. This search was limited to sequences belonging to the ITS region from fungi type and reference material. Taxonomic identification was limited to genus rank because often occurs that ITS sequences are not accurate for species rank identification (i.e. Ref.^30^). When the identity between our sequences and that of a type strain was lower than 95%, the genus of our sequence was considered to be unknown. Further taxonomic information was obtained by means of a phylogenetic tree made with representative sequences from each taxon. This tree was made with MEGA6 software using the maximum likelihood method with distances calculated according to the Tamura 3 parameter model^63^. Tree branch confidence values were estimated by bootstrapping with 1000 replications.

For each kale accession, the incidence of each fungal taxon in plants was calculated, relating the number of plants in which it was found against the total of plants sampled (30 root fragments per plant, 6 plants per accession, 30 plants in total), as alpha diversity analysis. Additionally, the distribution of the relative abundance of each taxon was observed with a rank-abundance curve. Furthermore, beta diversity was analyzed by comparing between accessions, using the Jaccard index.

### *In planta* assays

#### Plant–fungus interaction

One of the local varieties previously used in the diversity study (MBG-BRS0106, from here on referred to as kale) was used for the reinoculation tests and the study of the biological activity of the selected fungi.

Different fungal isolates (Table 3) were chosen based on their presence in all accessions, or their proven biological activity in other plant species (bibliographic search). To inoculate plants, one part of beet pulp inoculum was mixed with seven parts (w/w) of a substrate consisting of peat moss (Profi-Substract, Gramoflor, Valencia, Spain) previously treated at 80 °C for 24 h. The fungal inoculum was a 4-week-old culture of each fungus grown in sugar beet pulp medium^64^. Uninoculated controls were transplanted to soil mixed with uninoculated beet pulp medium. By qPCR, the ability of fungi to effectively colonize kale roots could be verified (data not shown).

**Table 3:** Fungal isolates used in kale inoculation.

Proposed taxon	Name in the text	Abbreviation	Presence in kale accessions
*Pleosporales* sp. B (next to *Pyrenophora*)	*Pyrenophora*	P	MBG-BRS0292 MBG-BRS0446 MBG-BRS0468
*Chaetomium* sp.	*Chaetomium* sp.	CG	MBG-BRS0292
*Trichoderma* sp.	*Trichoderma* sp.	TH	MBG-BRS0446
Helotiales sp. (next to *Phialocephala*)	*Phialocephala*	PH	MBG-BRS0426 MBG-BRS0468
*Pleosporales* sp. A (next to *Setophoma* and *Edenia*)	*Setophoma*/*Edenia*	ST	All
*Diaporthe* sp.	*Diaporthe* sp.	D	MBG-BRS0106
*Fusarium* sp.	*Fusarium* sp.	FO	All
*Acrocalymma* sp.	*Acrocalymma* sp.	AV	MBG-BRS0426 MBG-BRS0292 MBG-BRS0468
*Curvularia* sp.	*Curvularia* sp.	CB	MBG-BRS0426

#### Growth promotion

For the analysis of plant growth promotion kale plants were inoculated in 20 L pots. The plants were kept under controlled greenhouse conditions until their aerial part was harvested in 8-week-old-plants to record fresh weight. The plants were watered 2–3 times a week, according to the observed needs, always with the same amount of water in all the plants. Exogenous fertilization was not used. Greenhouse conditions were 14 h photoperiod, environmental temperature (12–30 °C) and relative humidity above 80%. A total of 25 plants were used for each fungal isolate inoculated.

#### Cold tolerance

For the cold tolerance assay, we stablished the same conditions reported in Ref.^65^. Seeds of kale were planted in 1 L pots and grown under fluorescent light (228 μmol m^−2^ s^−1^) in a 14 h light/10 h dark photoperiod regime and watered as needed. The temperature in the cold-exposure treatment was set at 12 ± 1 °C, since lower temperatures reduced seed germination and seedling survival dramatically. A total of 25 plants were used for each inoculation.

#### Biotic resistance

Inoculation of kale leaves with the bacterial plant pathogen *Xanthomonas campestris* pv. *campestris* (*Xcc*) was made according to Ref.^66^. Bacterial cultures were grown in PDA at 30 °C for 48 h. A bacterial suspension was diluted with sterile water to reach a turbidity value of 0.5, which corresponds to a concentration of 5 × 10^8^ cfu/mL. Turbidity of the suspension was measured with a microplate spectrophotometer (Spectra MR; DynexTechnologies, Chantilly, VA) at a wavelength of 600 nm. The second leaf counting from the apex was inoculated when plants were at a 5–6 leaf stage. Inoculation was made by biting on three veins located on the edge of the leaf with a mouse-tooth forceps wrapped in cotton submerged in the inoculum. *Xcc* race 1 type strain HRI3811, provided by Joana Vicente (University of Warwick, UK), was used to inoculate the plants. After the inoculation, plants were kept under controlled greenhouse conditions (20 °C at night and 28 °C during the day, and relative humidity greater than 80%). Damage was measured using a visual damage index (DI) scale ranging from 1 to 9, where 1 = no visible symptoms and 9 = severely diseased with typical V-shaped chlorotic leaf edge lesions presenting blackened veins areas. Data were taken 8 and 11 days post infection (d.p.i.). A total of 10 plants were used for each fungal strain.

Infestation with the insect pest *Mamestra brassicae* (*Mb*) was carried out under the controlled greenhouse conditions above described using 12-week-old-plants. Eggs were provided by the Laboratory of Entomology, Wageningen University, The Netherlands. Egg hatching larvae were fed with fresh leaves of *B. oleracea* (MBG-BRS0106) ad libitum. *Mb* inoculation was carried out depositing 5 7–10 days old larvae on the 5–6th true leaf of the plant. After 5 days, data on leaf area consumed per plant and the damage index were recorded: 1 = no damage, 2 = 1–10% leaf consumed, 3 = 11–20% leaf consumed, 4 = 21–30% leaf consumed, 5 = more than 30% of the leaf consumed. A total of 10 plants were used for each fungal strain.

### Statistical analysis

For each trait (i.e. growth promotion, tolerance to cold, inoculation with *Xcc* and infestation with Mb) a Student’s t-test was used to compare the means of each fungal inoculation treatment with the control at P < 0.05 and P < 0.01; significant differences are denoted using asterisks.

## Supplementary information

Supplementary Figure S1.
