# Supplementary figures and images for: Brassica oleracea var. acephala (kale) improvement by biological activity of root endophytic fungi
Source: Sci Rep. 2020 Nov 19;10:20224. doi: 10.1038/s41598-020-77215-7 (PMC7678862; doi:10.1038/s41598-020-77215-7)

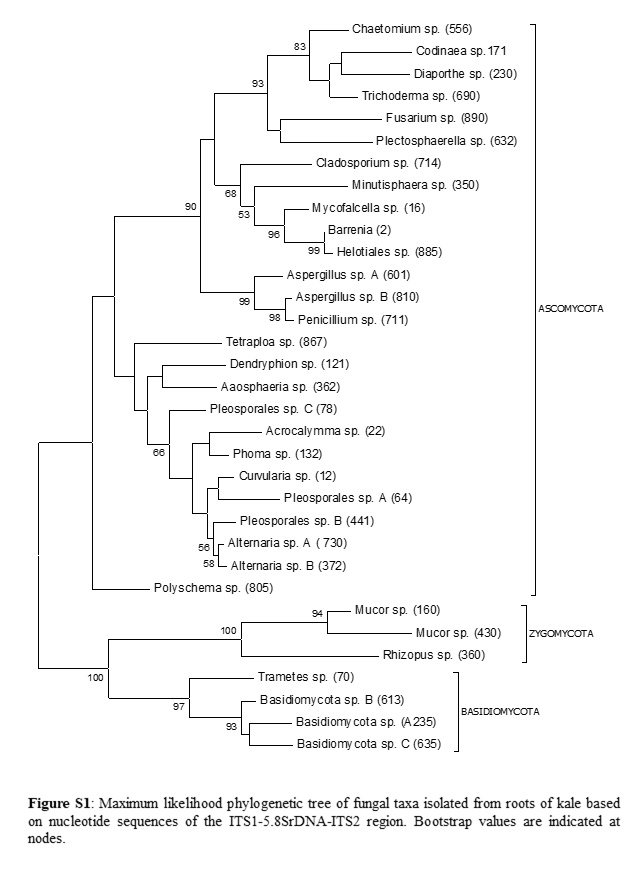

Supplement: Supplementary file 1 — Supplementary Figure S1. [file 41598_2020_77215_MOESM1_ESM.tif]
